# *In Silico* Analysis of Microarray-Based Gene Expression Profiles Predicts Tumor Cell Response to Withanolides

**DOI:** 10.3390/microarrays1010044

**Published:** 2012-05-22

**Authors:** Thomas Efferth, Henry Johannes Greten

**Affiliations:** 1 Department of Pharmaceutical Biology, Institute of Pharmacy and Biochemistry, Johannes Gutenberg University, Staudinger Weg 5, Mainz 55128, Germany; 2 Heidelberg School of Chinese Medicine, Karlsruher Straße 12, Heidelberg 69126, Germany; Email: heidelbergschool@aol.com; 3 Biomedical Sciences Institute Abel Salazar, University of Porto, Porto 4050-313, Portugal

**Keywords:** cancer, drug development, medicinal food, microarray, pharmacogenomics, natural products

## Abstract

*Withania somnifera* (L.) Dunal (Indian ginseng, winter cherry, Solanaceae) is widely used in traditional medicine. Roots are either chewed or used to prepare beverages (aqueous decocts). The major secondary metabolites of *Withania somnifera* are the withanolides, which are C-28-steroidal lactone triterpenoids. *Withania somnifera* extracts exert chemopreventive and anticancer activities *in vitro* and *in vivo.* The aims of the present *in silico* study were, firstly, to investigate whether tumor cells develop cross-resistance between standard anticancer drugs and withanolides and, secondly, to elucidate the molecular determinants of sensitivity and resistance of tumor cells towards withanolides. Using IC_50_ concentrations of eight different withanolides (withaferin A, withaferin A diacetate, 3-azerininylwithaferin A, withafastuosin D diacetate, 4-B-hydroxy-withanolide E, isowithanololide E, withafastuosin E, and withaperuvin) and 19 established anticancer drugs, we analyzed the cross-resistance profile of 60 tumor cell lines. The cell lines revealed cross-resistance between the eight withanolides. Consistent cross-resistance between withanolides and nitrosoureas (carmustin, lomustin, and semimustin) was also observed. Then, we performed transcriptomic microarray-based COMPARE and hierarchical cluster analyses of mRNA expression to identify mRNA expression profiles predicting sensitivity or resistance towards withanolides. Genes from diverse functional groups were significantly associated with response of tumor cells to withaferin A diacetate, e.g. genes functioning in DNA damage and repair, stress response, cell growth regulation, extracellular matrix components, cell adhesion and cell migration, constituents of the ribosome, cytoskeletal organization and regulation, signal transduction, transcription factors, and others.

## 1. Introduction

Drug resistance and severe adverse side-effects are major obstacles to cancer chemotherapy. Therefore, new therapy options with improved efficacy are urgently required. Nutritional sources such as marine and terrestrial plants are fertile grounds in which to find bioactive constituents with anti-tumor activity. The long-lasting experience of traditional phytotherapy may facilitate the identification of novel treatment strategies. In India, herbs have been used as foods and medicine for millennia. In recent years, the active principles of food and medicinal herbs have been increasingly elucidated, making the active chemical compounds accessible to molecular biological and biochemical research [1,2,3,4,5,6,7,8]. As compounds of *Ayurveda* (Sankskrit: knowledge of life) may have molecular targets different from those of standard anti-cancer drugs, they are attractive candidates in the search for novel drugs suitable to treat otherwise drug-resistant tumors. These natural compounds may also show reduced side effects on normal organs [9].

*Withania somnifera* (L.) Dunal (Indian ginseng, winter cherry, Sanskrit: *Ashwagandha*) belongs to the family of Solanaceae (nightshade plants). In Ayurveda, roots of the plant are either chewed or used to prepare beverages (aqueous decocts). Withania is widely known for its aphrodisiacal, liver tonic, anti-inflammatory, immune-stimulatory, and astringent activities. Furthermore, *Withania somnifera* is used to treat asthma, ulcers, emaciation, insomnia, senile dementia, tumors, diabetes, neurodegenerative disorders and numerous other symptoms and disorders [10,11,12,13,14].

The major phytochemicals in *Withania somnifera* and other *Withania* species are the withanolides, C-28-steroidal lactone triterpenoids built on an intact or rearranged ergostane framework. Within this framework, C-22 and C-26 are appropriately oxidized forming a six-membered lactone ring [15]. Other bioactive constituents in *Withania somnifera* are alkaloids, such as isopelletierine and anaferine [16].

In addition, *Withania somnifera*’s use in traditional medicine, *Withania somnifera* extracts as well as isolated withaferin A can exert chemopreventive effects [17,18,19,20]. Both substances have been shown to inhibit tumor cell growth in cell lines *in vitro*, in mouse tumors *in vivo*, and in human xenograft tumors transplanted into nude mice [21,22,23,24,25,26].

As is frequently observed with natural products in general, withaferin A exerts its anti-cancer effect by targeting multiple pathways rather than a single target in tumor cells. Withaferin A induces cell cycle arrest in the G_2_M phase [23,24,27,28,29,30]. Furthermore, withaferin A inhibits angiogenesis [31,32]. The full range of mechanisms contributing to the anti-cancer activity of withaferin A is incompletely understood at present. In addition, the cytotoxic and anti-cancer effects of other withanolides have not yet been thoroughly investigated either [33,34,35,36,37,38,39].

The aims of the present study were, firstly, to investigate whether tumor cells develop cross-resistance between standard anticancer drugs and withanolides and, secondly, to elucidate the molecular determinants of sensitivity and resistance of tumor cells to withanolides. Using IC_50_ concentrations of withanolides and the most frequently used standard clinical anticancer drugs, we analyzed the cross-resistance profile of 60 cell lines of the National Cancer Institute (NCI), USA [40]. Next, we performed transcriptomic microarray-based COMPARE analyses of mRNA expressions and then subjected the candidate genes to hierarchical cluster analyses to identify mRNA expression profiles, which predict sensitivity and resistance of tumor cell withanolides. Our microarray-based investigation resulted in novel candidate genes associated with the response of cancer cells to withaferin A diacetate. We found that tumor cell response was associated with genes from diverse functional groups (DNA damage and repair, stress response, cell growth regulation, extracellular matrix components, cell adhesion and cell migration, constituents of the ribosome, cytoskeletal organization and regulation, signal transduction, transcription factors, and others), indicating that resistance and sensitivity may be determined by multiple mechanisms.

## 2. Results

### 2.1. Cytotoxicity of Withanolides towards Cancer Cells

**Figure 1:**
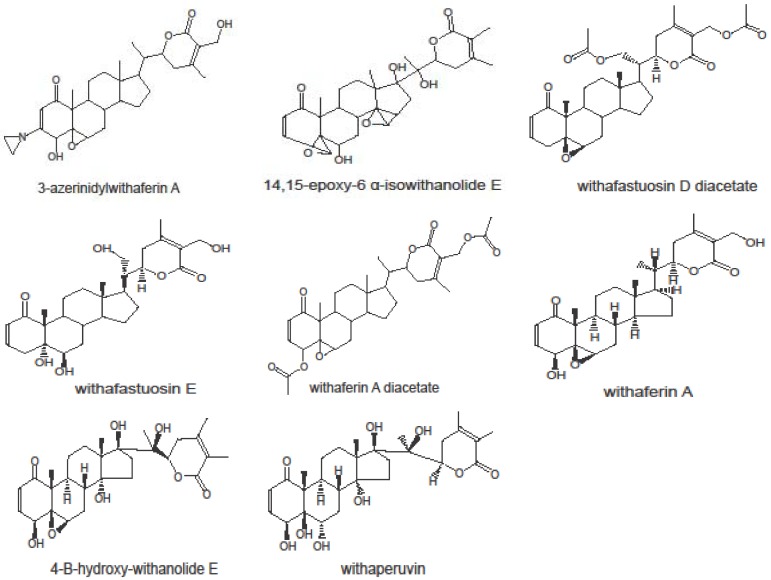
Chemical structures of the 8 withanolides tested.

**Figure 2:**
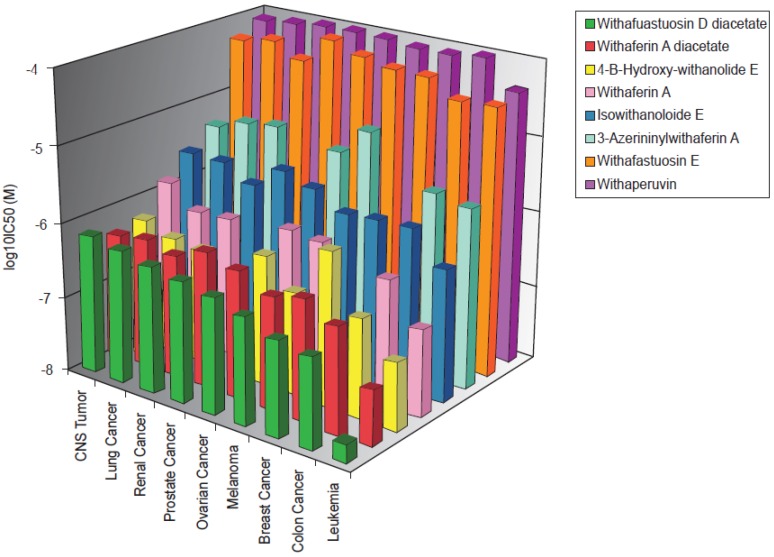
Mean log_10_IC_50_ values of 8 withanolides for tumor cell lines from the NCI drug screening panel as assayed by the sulforhodamine B test.

Eight withanolides were analyzed. Their chemical structures are shown in Figure 1. These substances were investigated over doses ranging from 10^−8^ to 10^−4^ M in 60 cell lines of the NCI, and log_10_IC_50_ values were calculated for each withanolide for each cell line. The mean log_10_IC_50_ values for cell lines of each tumor type are depicted in Figure 2. Withafastuosin D diacetate, withaferin A diacetate, and 4-B-hydroxy-withanolide E were the most cytotoxic compounds of the panel tested, whereas withafastuosin E and withaperuvin showed only minimal inhibitory activity towards the cancer cell lines in the panel. Withaferin A, isowithanololide E, and 3-azerininylwithaferin A demonstrated intermediate cytotoxicity (Figure 2). Among a panel of 60 tumor cell lines, leukemia cell lines were on average most sensitive towards withafastuosin D diacetate, withaferin A diacetate, 4-B-hydroxy-withanolide E, and withaferin A. Colon cancer lines were on average most sensitive among cell lines derived from solid cancers.

### 2.2. Cross-Resistance of the NCI Cell Line Panel Towards Withanolides

In order to find out whether the cell lines in the NCI panel exhibit cross-resistance to the eight withanolides investigated, we correlated the corresponding log_10_IC_50_ values to each other for each cell line. As shown in Table 1, correlation coefficients of R > 0.6 (Pearson’s correlation test) were found frequently for correlation among the withanolides, indicating that the cell line panel shows significant cross-resistance towards the various withanolides. Next, the log_10_IC_50_ values of the NCI cell line panel for the eight withanolides were correlated with those for established anticancer agents. Representative drugs from several major cancer drug classes (alkylating agents, platinum compounds, DNA topoisomerase I and II inhibitors, antimetabolites, and mitotic spindle poisons) were chosen. Significant correlations were consistently observed between withanolides and carmustine (BCNU), lomustine (CCNU), and semustine (methyl-CCNU), indicating that many of the NCI cell lines demonstrate cross-resistance between withanoloides and these nitrosoureas. The correlations between withanolides and other established anticancer drugs were weak (R < 0.6) (Table 1).

**Table 1:** Cross-resistance of the NCI cell line panel towards withanolides and anticancer drugs.

Compounds	R-/P-Values	Withafastuosin D diacetate	4-B-hydroxy-withanolide E	Withaferin A diacetate	Withaferin A	Isowithanololide E	3-Azerininyl-withaferin A	Withafastuosin E	Withaperuvin
**Withanolides:**									
Withafastuosin D diacetate	R-value		0.517	0.853	0.67	0.608	0.528	0.413	0.544
	P-value		1.00 × 10^−4^	9.18 × 10^−18^	4.37 × 10^−7^	2.08 × 10^−7^	8.22 × 10^−5^	8.64 × 10^−4^	5.11 × 10^−6^
4-B-hydroxy-withanolide E	R-value			0.548	0.548	0.452	0.42	0.492	0.469
	P-value			3.29 × 10^−5^	1.05 × 10^−4^	7.06 × 10^−4^	0.003	3.01 × 10^−4^	4.48 × 10^−4^
Withaferin A diacetate	R-value				0.701	0.7	0.475	0.426	0.623
	P-value				5.95 × 10^−8^	3.47 × 10^−10^	3.69 × 10^−4^	5.98 × 10^−4^	6.99 × 10^−8^
Withaferin A	R-value					0.609	0.549	0.358	0.466
	P-value					5.70 × 10^−6^	8.41 × 10^−5^	0.001	7.17 × 10^−4^
Isowithanololide E	R-value						0.348	0.439	0.513
	P-value						0.008	3.94 × 10^−4^	1.63 × 10^−5^
3-Azerininyl-withaferin A	R-value							0.28	0.349
	P-value							0.033	0.008
Withafastuosin E	R-value								0.291
	P-value								0.016
**Alkylating agents:**									
Carmustine (BCNU)	R-value	0.607	0.466	0.747	0.508	0.59	0.393	0.411	0.721
	P-value	1.74 × 10^−7^	4.21 × 10^−4^	5.64 × 10^−12^	2.16 × 10^−4^	4.47 × 10^−7^	0.003	8.27 × 10^−4^	5.79 × 10^−11^
Lomustine (CCNU)	R-value	0.515	0.509	0.579	0.517	0.43	0.304	0.381	0.479
	P-value	1.51 × 10^−5^	1.09 × 10^−4^	7.78 × 10^−7^	1.65 × 10^−4^	3.37 × 10^−4^	0.019	0.002	6.15 × 10^−5^
Semustine (me-CCNU)	R-value	0.542	0.389	0.653	0.381	0.519	0.305	0.362	0.618
	P-value	5.63 × 10^−6^	0.003	1.36 × 10^−8^	n.s.	1.48 × 10^−5^	0.019	0.003	1.2 × 10^−7^
Melphalan	R-value	0.471	0.268	0.496	0.349	0.429	0.186	0.253	0.55
	P-value	8.47 × 10^−5^	0.033	3.25 × 10^−5^	0.01	3.53 × 10^−4^	n.s.	0.03	3.24 × 10^−6^
Ifosfamide	R-value	0.162	0.222	0.14	0.265	0.136	0.204	0.199	0.083
	P-value	n.s.	n.s.	n.s.	0.041	n.s.	n.s.	n.s.	n.s.
**Platin compounds:**									
Cisplatin	R-value	0.199	0.11	0.296	0.191	0.282	0.05	0.131	0.379
	P-value	n.s.	n.s.	0.011	n.s.	0.015	n.s.	n.s.	0.002
Carboplatin	R-value	0.124	0.22	0.279	0.195	0.308	0.11	0.127	0.391
	P-value	n.s.	n.s.	0.016	n.s.	0.009	n.s.	n.s.	0.001
**DNA topoisomerase I inhibitors:**									
Camptothcin	R-value	0.205	−0.018	0.131	0.076	0.05	0.125	0.069	0.307
	P-value	n.s.	n.s.	n.s.	n.s.	n.s.	n.s.	n.s.	n.s.
Topotecan	R-value	0.242	0.119	0.178	0.108	0.102	0.104	0.198	0.308
	P-value	0.032	n.s.	n.s.	n.s.	n.s.	n.s.	n.s.	0.009
**DNA topoisomerase II inhibitors:**									
Adriamycin	R-value	0.327	0.198	0.362	0.203	0.196	0.267	0.207	0.413
	P-value	0.006	n.s.	0.002	n.s.	n.s.	0.035	n.s.	5.70 × 10^−4^
Daunorubicin	R-value	0.44	0.213	0.447	0.305	0.21	0.333	0.247	0.46
	P-value	2.44 × 10^−4^	n.s.	1.95 × 10^−4^	0.022	n.s.	0.011	0.033	1.25 × 10^−4^
Etoposide	R-value	0.237	0.063	0.244	0.256	0.199	0.15	0.109	0.246
	P-value	0.036	n.s.	0.031	0.047	n.s.	n.s.	n.s.	0.03
Teniposide	R-value	0.343	0.215	0.358	0.217	0.203	0.264	0.185	0.437
	P-value	0.004	n.s.	0.003	n.s.	n.s.	0.037	n.s.	2.67 × 10^−4^
**Antimetabolites:**									
5-Fluorouracil	R-value	0.357	0.16	0.365	0.054	0.3	0.25	0.272	0.129
	P-value	0.003	n.s.	0.002	n.s.	0.011	0.046	0.021	n.s.
Methotrexate	R-value	0.451	0.114	0.418	0.154	0.27	0.299	0.218	0.21
	P-value	1.66 × 10^−4^	n.s.	4.94 × 10^−4^	n.s.	0.019	0.021	n.s.	n.s.
**Mitotic spindle poisons:**									
Vincristine	R-value	0.283	0.266	0.307	0.177	0.102	0.446	0.258	0.156
	P-value	0.015	0.034	0.009	n.s.	n.s.	8.32 × 10^−4^	0.028	n.s.
Vinblastine	R-value	0.033	−0.222	0.041	−0.040	0.017	−0.007	0.085	0.058
	P-value	n.s.	n.s.	n.s.	n.s.	n.s.	n.s.	n.s.	n.s.
Paclitaxel	R-value	0.283	0.193	0.308	0.143	0.048	0.399	0.304	0.315
	P-value	0.015	n.s.	0.009	n.s.	n.s.	0.003	0.011	0.008
Taxotere	R-value	0.202	−0.041	0.158	0.146	0.098	0.337	0.177	0.215
	P-value	n.s.	n.s.	n.s.	n.s.	n.s.	0.01	n.s.	n.s.

n.s., not significant (P > 0.05).

### 2.3. COMPARE and Cluster Analyses of Microarray-Based mRNA Hybridization:

COMPARE analyses were performed to obtain a gene expression profile and identify the most up- or down-regulated genes correlated with the IC_50_ values for the withanolides. We performed COMPARE analyses of log_10_IC_50_ values for the three most cytotoxic withanolides (withafastuosin D diacetate, withaferin A diacetate, and 4-B-hydroxy-withanolide E) and the transcriptomic mRNA-based expression profiles of the NCI cell lines to produce scale indices of correlation coefficients. The microarray data from the NCI website [40] was used to perform further *in silico* analysis. The mRNA expression levels were determined by microarray analysis [41,42,43]. We performed a standard COMPARE analysis in which cell lines most inhibited by withanolides (lowest log_10_IC_50_ values) were correlated with the lowest mRNA expression levels of genes. These genes can be considered possible candidate genes in determining cellular resistance to withanolides. Furthermore, reverse COMPARE analysis was carried out, correlating the most inhibited cell lines with the highest gene expression levels. Considering a COMPARE coefficient of R > 0.6 as cut-off value, only two genes each fulfilled this criterion in connection with withafastuosin D diacetate and 4-B-hydroxy-withanolide E. These two compounds were, therefore, excluded from further analyses. The genes whose mRNA expression correlated with withaferin A diacetate are shown in Table 2. Table 2 differs from Table 1 in that it is rearranged in such a way that genes are grouped according to their order in the cluster analysis. This allows one to see which genes were clustered together and which ones were separated. Three main clusters were observed; however, a pattern of genes with similar functions was not seen among the clusters.

Among the genes were genes from diverse functional groups, such as DNA damage response and repair (*RAD54L*), stress response (*ANXA2*, *PPIH*, *UACA*), cell growth regulation (*BCAR3*, *CD53*, *NASP*, *TRIM3*), extracellular matrix components, cell adhesion and migration (*ADAM9*, *ASAP2*, *ITGB1*, *LAMB1*), ribosomal proteins (*RPS23*, *RPL5*, *LOC440055*, *LOC729362*), cytoskeletal organization and regulation (*CORO1A*, *LCP1*, *PLS3*, *WAS*), signal transduction (*ASAP2*, *BCAR3*, *DLG2*, *GNA11*, *PTPN7*, *RGS12, RNF138*, *SH3BP4*, *TJP1*), transcription factors (*IFZF1*, *HCLS1*, *TRIM3*, *ZNF112*, *ZNF228*),and others(*ALDH7A1*, *LSM2*, *MANBAL*, *NACA*, *STMN4*).

**Figure 3:**
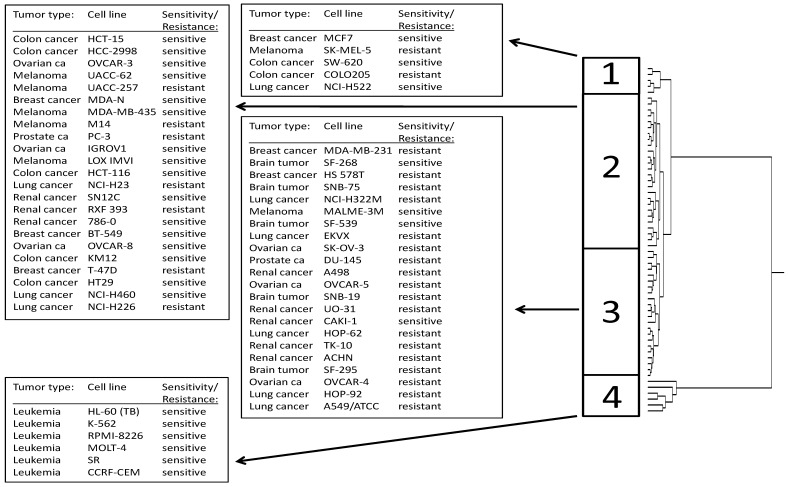
Dendrogram of hierarchical cluster analysis (complete linkage method) obtained from mRNA expression of 40 genes correlated with log_10_IC_50_ values for withaferin A diacetate. The dendrogram shows the clustering of 60 cell lines according to the mRNA expression profile for genes identified by COMPARE analyses (Table 3).

Next, the 40 genes identified by standard and reverse COMPARE analyses were subjected to hierarchical cluster analysis. The dendrogram obtained by this procedure can be divided into three major branches (Figure 3). The distribution of cell lines sensitive or resistant to withaferin A diacetate varies significantly between the branches of the dendrogram. The distribution of cell lines among the dendrogram predicts resistance to withaferin A diacetate with statistical significance (P = 0.00208 × 10^−^^6^; χ^2^-test; Table 3). 

**Table 2:** Correlation of constitutive mRNA expression of genes identified by compare analyses with IC_50_ values for withaferin A diacetate of 60 tumor cell lines.

Symbol	COMPARE Coefficient	ID No.	Genebank	Name	Function
**Cluster 1:**					
CORO1A	0.65	GC9728	AA047478	Coronin, actin binding protein, 1A	Cytoskeleton component
LSM2	0.646	GC31813	AJ245416	LSM2 homolog, U6 small nuclear RNA associated (S. cerevisiae)	Pre-mRNA splicing
HCLS1	0.676	GC34797	X16663	Hematopoietic cell-specific Lyn substrate 1	Transcription factor
**Cluster 2:**					
unknown	0.643	GC34785	X79234	unknown	Unknown
PLS3	−0.606	GC37799	M22299	Plastin 3	Actin-bundling protein
RAD54L	0.654	GC32858	X97795	RAD54-like (S. cerevisiae)	DNA repair and mitotic recombination
RPL5	0.648	GC36655	U14966	Ribosomal protein L5	Structural constituent of ribosome
IKZF1	0.648	GC61547	AI247840	IKAROS family zinc finger 1 (Ikaros)	Transcriptional regulator
DLG2	−0.612	GC10718	R41930	Discs, large homolog 2 (Drosophila)	Signal transducer, required for perception of chronic pain through NMDA receptor signaling
RPS23	0.669	GC37806	D14530	Ribosomal protein S23	Structural constituent of ribosome
unknown	0.674	GC33814	D11327	unknown	Unknown
RNF138	0.688	GC67595	AI608790	Ring finger protein 138	ubiquitin-protein ligase, ubiquitinylation
unknown	0.663	GC31615	X79234	unknown	Unknown
LCP1	0.649	GC27422	J02923	Lymphocyte cytosolic protein 1 (L-plastin)	Actin-binding protein, T-cell activation
LAMB1	−0.610	GC18026	AA004918	Laminin, beta-1	Extracellular matrix structural constituent
SH3BP4	−0.608	GC16071	W72796	SH3-domain binding protein	Signal transducer, functions in transferrin receptor internalization at the plasma membrane
UACA	−0.624	GC14684	N66980	Uveal autoantigen with coiled-coil domains and ankyrin repeats	Regulation of stress-induced apoptosis
BCAR3	−0.636	GC14433	N48319	Breast cancer anti-estrogen resistance 3	Adapter protein for activated growth factor receptors to signaling pathways that regulate cell proliferation
ZNF112	−0.640	GC15668	W15410	Zinc finger protein 112 homolog (mouse)	DNA binding, transcriptional regulator
LOC440055	0.721	GC36107	AA977163	Similar to ribosomal protein S12	Unknown
Unknown	−0.601	GC14769	N92652	unknown	Unknown
ALDH7A1	−0.642	GC16889	AA024918	Aldehyde dehydrogenase 7 family, member A1	Aldehyde dehydrogenase (NAD), oxidoreductase
ADAM9	−0.611	GC15762	W47533	ADAM metallopeptidase domain 9	Mediates cell-cell or cell-matrix interactions
TRIM3	−0.616	GC14991	N71362	Tripartite motif-containing 3	Transcriptional repressor, control of cell proliferation
ITGB1	−0.638	GC19072	AA044261	Integrin, beta 1 (fibronectin receptor, beta polypeptide, antigen CD29 includes MDF2, MSK12)	integrin binding
RGS12	−0.639	GC15931	W67134	Regulator of G-protein signaling 12	Signal transducer, regulator of G proteins
TJP1	−0.612	GC12455	R79560	Tight junction protein 1 (zona occludens 1)	Signal transduction for tight junction assembly and stabilizing junctions
ASAP2	−0.638	GC15131	N70773	ArfGAP with SH3 domain, ankyrin repeat and PH domain 2	GTPase activator, Regulates the formation of post-Golgi vesicles, modulates cell migration
STMN4	−0.650	GC11515	H29581	Stathmin-like 4	Unknown
WAS	0.654	GC69113	AI655719	Wiskott-Aldrich syndrome (eczema-thrombocytopenia)	effector for Rho-type GTPases, regulates structure and dynamics of the actin cytoskeleton
NACA	0.652	GC30164	AF054187	Nascent polypeptide-associated complex alpha subunit	Prevents inappropriate targeting of non-secretory polypeptides to the endoplasmic reticulum (ER)
NASP	0.657	GC83792	AW003362	Nuclear autoantigenic sperm protein (histone-binding)	Involved in DNA replication, normal cell cycle progression and cell proliferation
LOC729362	0.679	GC31589	T89651	Similar to ribosomal protein L36a	Unknown
GNA11	−0.639	GC31915	N36926	Guanine nucleotide binding protein (G protein), alpha 11 (Gq class)	Signal transducer, activator of phospholipase C, GTPase
unknown	−0.658	GC32458	M69013	unknown	Unknown
**Cluster 3:**					
CD53	0.666	GC89937	M37033	cell differentiation antigen 53,32-40kDa	Growth regulation in hematopoietic cells
PPIH	0.662	GC28763	AF016371	Peptidylprolyl isomerase H (cyclophilin H)	Chaperone
PTPN7	0.707	GC90165	M64322	Protein tyrosine phosphatase, non-receptor type 7	Regulation of T and B-lymphocyte development and signal transduction
ANXA2P3	−0.652	GC90123	M62895	Annexin A2 pseudogene 3	Unknown
ANXA2	−0.659	GC85483	D00017	Annexin A2	Phospholipase inhibitor, involved in heat-stress response

Only genes with correlation coefficients of >0.6 or <−0.6 were considered. Positive correlation coefficients indicate direct correlations to log_10_IC_50_ values; negative ones indicate inverse correlations. Information on gene functions was taken from the OMIM database, NCI, USA [44] and from the GeneCard database of the Weizman Institute of Science, Rehovot, Israel [45].

**Table 3:** Separation of clusters of 60 NCI cell lines obtained by hierarchical cluster analysis shown in Figure 3 in comparison to drug sensitivity. The median log_10_IC_50_ value (−6.5 m) for each compound was used as cut-off to separate tumor cell lines as being “sensitive” or “resistant”.

	Partition	Cluster 1	Cluster 2	Cluster 3	Cluster 4
sensitive	<−6.5	3	9	4	6
resistant	>−6.5	2	17	18	0
χ^2^-test	P = 0.00208				

In the present investigation, we showed that six out of the eight withanolides tested exerted profound cytotoxic activity towards tumor cell lines. The cytotoxicity of the withanolides was compared among tumor cell lines belonging to nine different tumor types. We found that leukemic cell lines were particularly sensitive against withanolides. This observation is consistent with previous results obtained by our group that indicate phytochemicals and standard anticancer drugs are frequently more cytotoxic to leukemia cells than to cell lines derived from solid tumors [46,47,48]. Among the cell lines of solid tumors, colon cancer cells were most sensitive to withaferin A diacetate. A possible explanation might be that the Notch-1 signaling pathway plays an important role in colon carcinogenesis and that withanolides inhibit Notch-1 signaling [49]. 

Our investigation confirms previous reports showing the cytotoxic activity of withanolides against cancer cells [21,22,24,25,26,28]. Furthermore, we have analyzed the cross-resistance profile of the cell line panel to different withanolides and between withanolides and established anticancer drugs. By means of Pearson’s correlation test, we found that the 60 cell lines exerted significant cross-resistance among withanolides. We observed that cell lines sensitive to one withanolide are likely to be sensitive to other withanolides and *vice versa*. 

We extended this analysis to standard anticancer drugs, and found consistent cross-resistance between withanolides and nitrosoureas. Cross-resistance to other drug classes (platin compounds, DNA topoisomerase I or II inhibitors, antimetabolites, mitotic spindle poisons) was less frequent or not observed. This suggests that withanolides and nitrosoureas may share similar mechanisms of action, e.g., DNA damage of tumor cells. Interestingly, withaferin A has been described as exerting chemo- and radiosensitizing effects on tumors *in vitro* and *in vivo* [9,50,51,52]. It is possible that the interaction of the activity of two treatment substances on DNA may lead to synergistic and sensitizing effects.

As a next step, we correlated the IC_50_ values of withaferin A diacetate on 60 tumor cell lines with the microarray-based transcriptomic mRNA expression levels of the cell line panel [53] by COMPARE analysis. This approach has been successfully used to unravel the mode of action of novel compounds [53]. Cluster and COMPARE analyses are also useful for comparing gene expression profiles with IC_50_ values for investigational drugs to identify candidate genes causing drug resistance [54] and to identify prognostic expression profiles in clinical oncology [55]. 

We identified genes from diverse functional groups that were significantly associated with the response of tumor cells to withaferin A diacetate. These genes were related to DNA damage and repair, stress response, cell growth regulation, extracellular matrix components, cell adhesion and cell migration, constituents of the ribosome, cytoskeletal organization and regulation, signal transduction, transcription factors, and others. 

The fact that genes associated with sensitivity or resistance against withaferin A diacetate were from diverse functional groups speaks for the multiplicity of mechanisms by which withaferin A diacetate inhibits cancer cells. This so-called “multiplicity of mechanisms” can refer to multiple targets that together lead to multiple effects or it can refer to one target leading to activation or inactivation of multiple downstream pathways. Multi-specificity is a general feature of many natural products; rather than acting on one single target, they affect multiple targets and pathways [8].

The multifaceted nature of withanolides has been previously recognized, e.g., induction of G2M cell cycle arrest [27] and apoptosis [34], inhibition of metastasis [56] and angiogenesis [57], inhibition of the transcription factor NFκB [58] and heat shock protein HSP90 [25,59] , and immunomodulation [20]. It is interesting that our transcriptomic approach pointed to additional mechanisms, whose role for response of tumor cells against withanolides have not been considered so far. 

For example, RAD54L is involved in recombinatorial DNA repair (via the RAD52 pathway) and dissociates RAD51 from nucleoprotein filaments formed on double-stranded DNA [45,60]. The cross-resistance profile of withanolides and nitrosoureas for the 60 cancer cell lines may be explained at least in part by RAD54L. This speculation deserves further investigation in the future. 

The significant correlation of the expression of stress response genes to the log_10_IC_50_ values for withanolides indicates that cell lines with high expression of these genes better resist the cytotoxic effects of withaferin A diacetate than cell lines with low expression levels. ANXA2 is a phospholipase inhibitor, which is involved in the heat stress response [45]. Heat shock proteins and chaperones are known to mediate resistance to conventional anticancer drugs [61]. The possibility of ANXA2 playing a role in multidrug resistance and gemcitabine resistance has been suggested [62,63]. PPIH accelerates protein-folding and may act as chaperone, and UACA regulates stress-induced apoptosis by NFκB inhibition [45]. PPIH and UACA have not yet been linked to cellular response to established drugs or withanolides. 

Several genes involved in cell growth regulation were significant in our analysis, namely, *CD53, BCAR3, TRIM3,* and *NASP*. Although these genes do not belong to the set of classical cell cycle genes, they suggest that withaferin A diacetate may play a role in inhibiting cancer cell proliferation. Many established anticancer drugs also act against cell proliferation. However, most classical anticancer drugs damage not only cancer cells, but normal proliferating cells, which leads to the severe side effects often observed with chemotherapy, e.g., myelosuppression, sterility, gastrointestinal mucosa damage, and alopecia. Whether withaferin A diacetate also exerts detrimental effects on normal tissues is not known and requires further exploration. 

Interestingly, a number of genes encoding extracellular matrix (ECM) components and genes involved in cell adhesion and migration correlated with log_10_IC_50_ values for withaferin A diacetate including *ADAM9, LAMB1, ITGB1, ASAP2*. The ECM, cell adhesion and migration are important components in cancer metastasis and progression. Withanolides are known to inhibit metastasis [56]. We suggest that these four genes identified in our analysis may contribute to the anti-metastatic activity of withaferin A diacetate. 

Another interesting finding is that cellular response to withaferin A diacetate was correlated with the expression of genes encoding several constituents of the ribosome. Whereas the role of ribosomal proteins in resistance towards established anticancer drugs has not yet been intensively investigated [64], our study indicates that the ribosomal genes *RPS23* and *RPL5* and the still poorly characterized ribosome-associated genes *LOC440055* and *LOC729362* affect resistance towards withaferin A diacetate. Ribosomal proteins are often involved in antibiotic resistance. For example, Streptomycin resistance is based on the modification of an aspartic acid moiety in the ribosomal protein S12 [65]. RPS6 is thought to play an important role in controlling cell growth and proliferation by selective translation of particular classes of mRNA [66]. The RPL6 gene plays an important role in the development of drug resistance in leukemia and gastric cancer cells by suppressing drug-induced apoptosis [64,67]. In previous investigations, we observed that genes encoding ribosomal proteins correlated to cellular sensitivity or resistance towards several cytotoxic phytochemicals, including shikonin, resibufogenin, and artesunate [6,68,69]. 

## 4. Experimental Section

*Statistical Analyses:* The panel of human tumor cell lines of the Developmental Therapeutics Program of NCI and their testing by sulforhodamine B assay and mRNA microarray hybridization have been described [42,70,71]. The data from these assays can be found at the NCI website [40]. For hierarchical cluster analysis, objects were classified by calculation of distances between individuals 445rfG, by means of the complete linkage method. All objects were assembled into cluster trees (dendrograms). Previously, cluster models were validated for gene expression profiling and for approaching molecular pharmacology of cancer [70,72]. Hierarchical cluster analyses applying the complete linkage method were performed with the WinSTAT program (Kalmia, Cambridge, MA, USA). Missing values were automatically omitted, and the closeness of any two joined objects was calculated by the number of data points they contained. In order to calculate distances between all variables included in the analysis, the program automatically standardizes the variables by transforming the data to a set of values with mean = 0 and variance = 1.

For COMPARE analysis, the mRNA expression values of genes of interest and log_10_IC_50_ values for the withanolides were selected from the NCI database [40]. mRNA expression was determined by microarray analyses as reported [70]. COMPARE analysis was performed to produce rank-ordered lists of genes expressed in the NCI cell lines. The methodology has been described previously in detail [73]. Briefly, every gene of the NCI microarray database was ranked for similarity of its mRNA expression to those under the log_10_IC_50_ concentration for the corresponding compound. To derive COMPARE rankings, a scale index of correlation coefficients (R-values) was created. In the standard COMPARE approach, greater mRNA expression in cell lines correlates with enhanced drug resistance, whereas in reverse COMPARE analyses, greater mRNA expression in cell lines indicates drug sensitivity. 

Pearson’s correlation test was used to calculate significance values and rank correlation coefficients as a relative measure of the linear dependency of two variables. This test was implemented into the WinSTAT Program (Kalmia). The Pearson correlation test was used as a measure for interval-scaled linear correlations. We used the Pearson test rather than the Spearman’s Rank correlation test because Spearman’s test is based on the equidistance of values, and the values used for our analysis were not equidistant.

The χ^2^-test was applied to bivariate frequency distributions of pairs of nominal scaled variables. This test was also implemented into the WinSTAT program (Kalmia Co.). The χ^2^-test determines the difference between each observed and theoretical frequency for each possible outcome, squares them, divides each by the theoretical frequency, and takes the sum of the results. Performing the χ^2^-test necessitated defining cell lines as being sensitive or resistant to withaferin A diacetate. This was done by taking the median IC_50_ value log_10_ = -6.5 M for withaferin A diacetate as a cut-off threshold.

## 5. Conclusions

In summary, our microarray-based investigation delivered novel candidate genes that were associated with the response of cancer cells to withaferin A diacetate. These results merit further investigation to prove the causative contribution of these genes to withaferin A diacetate resistance and sensitivity.
